# Prevalence and specificities of red cell alloantibodies among blood recipients in the Malaysian state of Kelantan

**DOI:** 10.4103/0973-6247.75997

**Published:** 2011-01

**Authors:** Fawwaz Al-Joudi, Anuar Bin Ali, Majdan Bin Ramli, Suhair Ahmed, Mohd Ismail

**Affiliations:** *Department of Biomedical Science, Faculty of Allied Health Sciences, National University of Malaysia, Kuala Lumpur, Malaysia*; 1 *Unit of Haematology, Hospital Raja Perempuan Zainab II, Kota Bharu, Malaysia*; 2 *Department of Haematology and Blood Transfusion, School of Medical Sciences, University of Science of Malaysia, Kota Bharu, Malaysia*; 3 *Department of Community Medicine, School of Medical Sciences, University of Science of Malaysia, Kota Bharu, Malaysia*

**Keywords:** RBC antigens, alloantibodies, blood transfusion

## Abstract

**Background:**

Red blood cell (RBC) alloantibodies may be formed following exposure to RBC antigens. In most cases, the alloimmunization develops during pregnancy or from previous blood transfusions. The RBC antigens and their alloantibodies vary among different human populations and ethnic groups, and they do have a clinical significance for their adverse immunological reactions.

**Aims:**

This study aimed at studying the prevalence of RBC alloantibodies at the Blood Transfusion Unit of Hospital Raja Perempuan Zainab II in Kota Bharu, Malaysia.

**Patients and Methods:**

A cross-sectional study was performed utilizing data obtained in the years 2007 and 2008. Data of antibody screening tests from 5719 patients were examined.

**Results and Discussion:**

The overall prevalence of alloimmunization was 65 (1.13%). The majority of these had a single alloantibody (76.9%), whereas the remaining 23.1% had multiple antibodies. The anti-E antibody comprised the most common alloantibody (24.6%) followed by the anti-Lewis (a) antibodies (18.5%) and the anti-M antibody (13.8%). There were more female recipients than males.

**Conclusions:**

It was concluded that the findings of this work have been comparable with other published works, and that the main factors associated with alloantibody formation were multiple transfusions and pregnancies. The study also emphasizes the necessity for carrying out immunohematology studies prior to every blood transfusion especially in cases that require multiple transfusions for a long period of time such as in thalassemia patients.

## Introduction

Many blood group antigens and their genes have been identified, and their physiological roles uncovered, and have been found to be important determinants in transfusion medicine. Approximately, 400 red blood cell (RBC) antigens have been identified. These RBC antigens and alloantibodies differ significantly among human populations and ethnic groups. Hence, alloimmunization after exposure to red cell alloantigens depends on genetic and acquired patient-related factors, dose, and the immunogenicity of the antigens.[1,2] The exact kinetics of alloimmunization are not clear.[3,4] The development of alloantibodies can significantlycomplicate transfusion therapy and result in difficulties in cross-matching of blood. Clinically, significant antibodies are capable of causing mild or severe adverse events following transfusion, such as hemolytic disease of the fetus and newborn. Thus, knowledge of such alloantibodies is essential for selecting appropriate RBC products for transfusion.[5] Antibodies that may cause hemolysis include those specific to most of the major and the minor blood groups.[3,5–9] One report on autoantibodies to red cells in thalassemia patients in Kelantan has been published.[10] This work was carried out at Hospital Raja Perempuan Zainab II, to determine the prevalence and distribution of RBC alloantibodies among blood recipients in Kelantan State.

## Materials and Methods

This is a retrospective cross-sectional study that utilized data of all patients admitted at Hospital Raja Perempuan Zainab II, Kota Bharu Kelantan during the years 2007 and 2008. Patients with missing data were excluded from the study. The essential data sought were those of alloantibodies obtained during routine screening and immunohematological investigations such as group, screen and hold (GSH) as part of the support of the transfusion service at the hospital. Other information sought for each patient included medical history, gender, age, and ethnic origin. Screening for antibodies utilized commercially prepared RBC test panels. In total, there were data from 5719 patients recruited in the study. The data were analyzed using the Statistical Package for the Social Sciences (SPSS) software Version 16.0.

## Results

**Figure 1:**
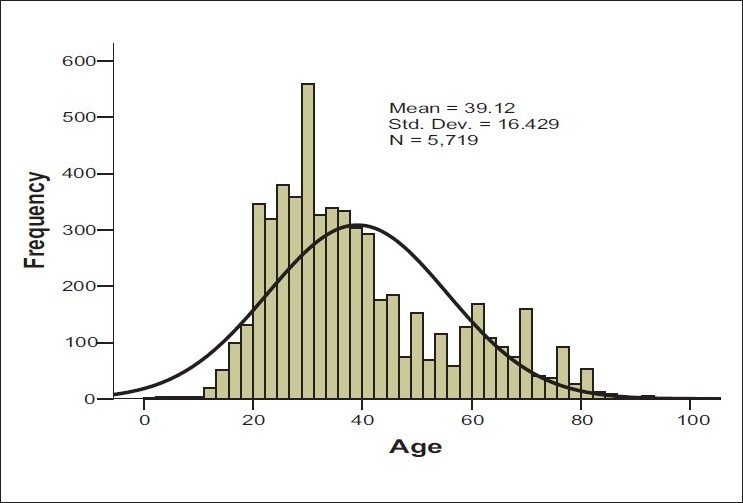
Histogram showing the age distribution of the study patients

**Figure 2:**
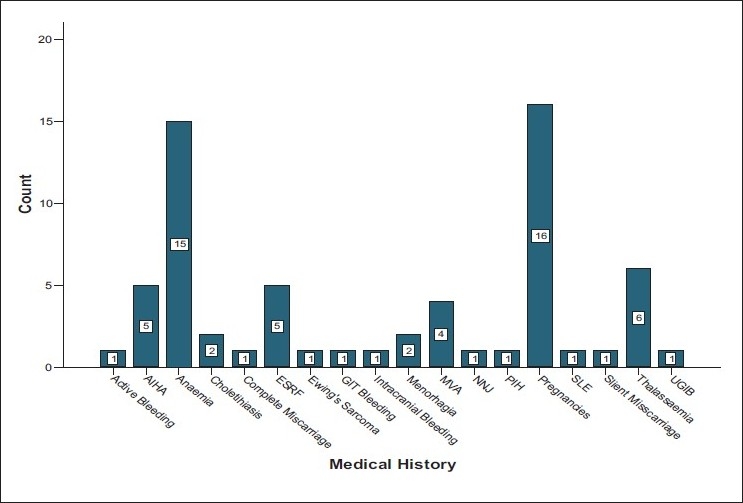
A bar chart showing the sample distribution according to their medical histories

The initial investigations showed that the majority of the recipients were female patients, most of whom were Malays, followed by Chinese, Siamese, Indians, and aboriginis, the Orang Asli. Their mean age was 39.12 ± 16.5 years old [Table 1 and Figure 1]. The medical histories were widely distributed, though dominated by pregnancies and anemias Figure 2. Of all the records investigated, only 65 patients (1.13%) were found to be positive for alloantibodies to RBC antigens (95% CI: 0.8-1.4). However, there were another 32 patients who harbored serum autoantibodies [Table 2]. The blood types records showed that 23 patients had blood group A, Rhesus positive (35.4 %), 21 patients had blood group B, Rhesus positive (32.3%), 17 patients with blood Group O, Rhesus positive (26.2 %), and 3 patients with blood group AB, Rhesus positive (4.6 %). In addition, there was one patient with blood group O, Rhesus negative 1 (1.5 %) [Table 3] of the 65 patients with alloantibodies, 50 patients (76.9%) had a single alloantibody, whereas 15 patients (23.1%) had multiple alloantibodies [Table 4]. Investigating the alloantibody specificities detected mostly Anti-E, Anti-Lewis (a), and Anti-M [Table 5].

**Table 1:** Descriptive analysis of study patients

Ethnicity	Gender		Total
	Female	Male	
Malay	3896	1613	5509 (96.3)
Chinese	88	64	152 (2.65)
Siamese	40	12	52 (0.91)
Indian	2	1	3 (0.05)
Orang Asli	3	0	3 (0.05)
Total	4029 (70.4)	1690	5719

Figures in parentheses are in percentage

**Table 2:** The prevalence of alloantibodies and autoantibodies among the study patients

Variable	*N*	Prevalence	95% CI
Alloantibody	65	1.13	0.8–1.4
Autoantibody	32	0.56	0.36–0.5

**Table 3:** The distribution of blood groups among the study patients

Type of blood group	Frequency (%)
O, Rh positive	17 (26.2)
A, Rh positive	23 (35.4)
B, Rh positive	21 (32.3)
AB, Rh positive	3 (4.6)
O, Rh negative	1(1.5)

**Table 4:** The distribution of RBC alloantibodies based on number of alloantibodies in each study subject (*n* = 65)

Variable	*N* (%)
Single	50 (76.9)
Multiple	15 (23.1)

**Table 5:** The distribution of RBC alloantibody types detected

Variable	Frequency *(n)*	Percentage
Anti-c	2	3.1
Anti-D	1	1.5
Anti-E	16	24.6
Anti-E + Anti-Jka	1	1.5
Anti-E + Anti-K + Auto-IgG	7	10.8
Anti-Jkb	2	3.1
Anti-Lewis (a)	12	18.5
Anti-Lewis (a + b)	7	10.8
Anti-Lewis (b)	7	13.8
Anti-M	9	10.8
Anti-E + Auto IgG	1	1.5
Total	65	100.0

The factors that associated with the development of RBC alloantibodies were also investigated. The total number of patients with complete data in their case records was 400. The variables tested showed that male patients had the odds ratio of 0.08 for developing alloantibody as compared to female patients. In addition, patients with histories of previous blood transfusions were 2.35 times more liable to developing alloantibodies compared to those with no histories of blood transfusions. Furthermore, blood group A-positive patients showed odds of developing alloantibodies as high as 12.21 when compared to patients with other blood groups [Table 6]. Moreover, female patients appeared to be significantly more liable to developing alloantibodies than male patients [Table 7]. The ethnic grouping showed that the prevalence of alloantibody associated stronger with the Malay group than with non-Malay groups [Table 8].

**Table 6:** The factors associated with the development of RBC alloantibody (*n* = 400)

Variable	n	Wald	*P* value	Odds	95% OR
		statistics		ratio	(CI)
		(df)		(OR)	
*Gender*					
Female (reference)	52	29.92(1)	<0.001	0.08	0.04–0.20
Male	348				
*History of transfusions*					
No (reference)	325	5.52 (1)	0.019	2.35	1.15–4.78
Yes	75				
*Blood groups*					
Other than A	364	34.99 (1)	<0.001	12.21	5.33–27.97
positive	36				
A+ ve					

Fitness of the model was tested by Hosmer and Lemeshow, P > 0.05.

**Table 7:** The association between frequency of alloantibody formation and gender

Gender	% of alloantibody n (%)		Chi square	*P* value
	Yes	No	value, χ^2^	
Male	43 (0.8%)	1647 (28.8%)	42.13	<0.001
Female	22 (0.4%)	4007 (70.1%)		

**Table 8:** The association of alloantibody with ethnicity

Ethnicity	% of alloantibody *n*		Chi square	*P* value
	Yes	No	value, χ^2^	
Malay	57 (1.0%)	5452 (95.3%)	13.86	<0.001
Non-Malay	8 (0.1%)	202(3.5%)		

## Discussion

The incidence of alloimmunization against RBC antigens depends on the demography of the population being studied. Previous data from a number of communities describe alloimmunization following transfusions for indications such as anemia, thalassemia, and end-stage renal failure (ESRF).[3,11–13] Such data and its related clinical indications were not available in Kelantan, Malaysia.

The overall prevalence of alloimmunization among blood recipients in this work is comparable with rates previously reported on patients receiving transfusion. This study shows that the majority of the study subjects have single rather than multiple alloantibodies of which anti-E was the most common alloantibody found followed by anti-Lewis (a) and anti-M, which may be determined genetically. The anti-E was detected in almost all available studies at relatively high levels. Others with somewhat widely distributed expression are the alloantibodies against anti-Lewis (a) and anti-Lewis (b). This remark suggests that anti-E and Lewis alloantibodies are the most common alloantibodies among populations. Furthermore, it implies that the E antigen and the Lewis (a and b) antigens are highly immunogenic and that they are expressed differentially among individuals of one community. In other words, the absence of antigen E may render a recipient prone to sensitization by the E antigen that comes from an E-positive donor.[14] This explanation marks the necessity for RBC phenotyping to stop unnecessary sensitization to RBC antigens, and to aid in avoiding unwanted clinical consequences.

In this study, as in most other studies, the incidence of alloimmunization among females is more predominant than in male patients, possibly because most of the blood recipients are females, especially those with histories of eventful pregnancies. Hence, immunization through pregnancy could be one main reason for the high incidence of RBC alloimmunization among female patients. However, female patients were reported not to be a majority once.[15] The ethnic distribution of alloantibodies indicates that Malays are predominantly affected, which is attributable to the fact that Kelantan State is populated by a great majority of Malays. Also expected, patients who had experienced blood transfusions were found to be more liable to developing alloantibodies than those who never experienced a blood transfusion. Similar findings have been indicated in other works.[12,16] However, the statistical association between the development of alloantibodies with blood group A was not clear. No such remark has been reported.

Nevertheless, this work represents a pilot study, which attempted to shed some light into the blood groups that have the potential for alloantibody formation. Hence, two things are recommended, knowledge of prevalent RBC antigens in a community and routine investigation for alloantibodies in blood donors.
